# Popularity of HIV self-tests may say more about the state of our primary care system than about the device itself

**DOI:** 10.14745/ccdr.v50i12da02

**Published:** 2024-12-05

**Authors:** Alexandra Musten, Patrick O’Byrne

**Affiliations:** 1 University of Ottawa, Ottawa, ON

**Keywords:** HIV/AIDS, primary care, self-test, STBBI

## Abstract

**Background:**

In Canada, HIV transmission continues to disproportionately affect the same communities of gay men, bisexual men and men who have sex with men (gbMSM); members of African, Caribbean or Black communities (ACB); people who use injection drugs; Indigenous people; and women who belong to the aforementioned groups. While primary care is an ideal location for HIV testing for members of these groups, many people do not have access to such healthcare services. In response, we launched GetaKit to distribute HIV self-tests.

**Methods:**

In light of reduced access to healthcare services as a result of the pandemic and in anticipation of Health Canada’s approval of an HIV self-test, a clinician-scientist research team at the University of Ottawa developed GetaKit: an online platform to provide access to sexual health services. When GetaKit first launched in Ottawa in July 2020 with funding from the Ontario Ministry of Health, its objectives were to ensure that access to the newly approved device remained 1) clinically appropriate, 2) accessible and 3) linked to care.

**Results:**

Over the course of the study, there were a stable number of individuals who reported having never been tested for HIV before. These individuals tended to be younger and more likely to be members of racialized minority groups; similar characteristics to those who also face the most barriers to primary care access.

**Conclusion:**

With new reports indicating that nearly six million Canadians are without a primary care provider, it was proposed that the popularity of the HIV self-test may tell more about this lack of access than about the utility of the device itself. While projects like GetaKit should be part of the broader strategy to overcome historic testing barriers, such as geographic distance and inconvenient clinic hours, it is important that this occurs in an environment where a strong primary care health system can support treatment, follow-up and specialist referrals, as required.

## Introduction

In Canada, primary care is often an individual’s first interaction with the healthcare system and is considered a basic tenet of universal health coverage ((1)). Yet, there are individuals in Ontario who are not connected with a primary care provider, resulting in unmet healthcare needs and worsened health outcomes ((2)). Moreover, during the COVID-19 pandemic, nearly 170,000 people in Ontario lost access to their primary care providers due to burnout and retirements ((3)). This is of particular concern with respect to HIV testing because, in Ontario, most HIV tests are ordered by primary care providers. The result of the foregoing situation is that, in 2020, HIV testing decreased by 56% in sexual health clinics, 42.8% in community health centres and 31.1% in other clinic settings ((4)).

Of further concern is that there is considerable overlap between the individuals who experience reduced access to primary care and the groups who are most affected by HIV in Ontario, including gay men, bisexual men and men who have sex with men (gbMSM); members of African, Caribbean or Black communities (ACB); people who use injection drugs; Indigenous people; and women who belong to the aforementioned groups ((2)). A randomized controlled trial performed in Ontario in 2021 found that physicians who had been practising over 20 years were nearly 13 times less likely to take on a patient who disclosed having an opioid use disorder ((5)). Black people also continue to be disproportionately affected by social and health inequities, including poor access to healthcare, and experience worse health outcomes ((6)). Another study reviewing hospitalization rates among Ontario’s Indigenous communities found that their higher rate of hospitalization compared to the general population is indicative of insufficient or ineffective primary care ((7)). In a setting where primary care is responsible for the delivery of prevention services and management of chronic diseases, and is a referral source to specialists, it is unacceptable that groups continue to experience vulnerability due to their sometimes deliberate exclusion from basic care ((7)).

## Methods

In light of reduced access to healthcare services as a result of the pandemic and in anticipation of Health Canada’s approval of an HIV self-test, a clinician-scientist research team at the University of Ottawa developed GetaKit: an online platform to provide access to sexual health services. When GetaKit first launched in Ottawa in July 2020 with funding from the Ontario Ministry of Health, its objectives were to ensure that access to the newly approved device remained 1) clinically appropriate, 2) accessible and 3) linked to care. Individuals who wished to obtain an HIV self-test were invited to review and accept the study consent form, create their account and complete the self-assessment questionnaire at GetaKit.ca. If testing was recommended, an HIV self-test was shipped to their address with linkage to care information. Participants were sent email reminders to submit their results by logging into their GetaKit.ca account. Individuals who submitted an invalid result were given a new test, individuals who submitted a negative result were sent a retest reminder three months later and those who submitted a positive result were provided support and then directly linked to confirmatory testing and care.

## Results

During the first six months of launching GetaKit.ca, 1,268 individuals visited the website and 47.3% (n=600) were eligible to receive a free HIV self-test. Of the 399 individuals who were eligible and completed the assessment, 71% (n=283) reported belonging to at least one of the groups that are most affected by HIV in Ontario, 24% reported no prior HIV testing and 33% (n=128) indicated that they did not have a primary care provider. For those who reported prior HIV testing, 55% (n=154) had been tested in a public health clinic and 34% had tested with a primary care provider. Interestingly, individuals who reported not being a member of the groups most affected by HIV in Ontario were nearly five times more likely to have been tested by a primary care provider compared to a public health clinic, underscoring access barriers among equity-deserving groups ((8)).

Since the first phase of GetaKit.ca, this service is now available across Ontario and, to date, has offered testing to over 17,000 people. Of these, 65% were cisgender male, 26% were cisgender female, 51% identified as gbMSM and 32% as heterosexual. Notably, 18% of GetaKit.ca participants identified as ACB, when ACB persons only make up 4% of Ontario’s population ((9)). GetaKit has furthermore identified 32 new HIV diagnoses since 2020, all of whom have completed confirmatory bloodwork and have been linked to care in their area. Over the course of the project, another observation is that a sizeable and stable number of participants (~30%) reported that this was their first time testing for HIV; an important indicator of success, as it demonstrates engagement with individuals who may not have accessed testing otherwise. First-time testers accessing GetaKit tended to be younger and more likely to be members of racialized minority populations ((10)).

## Discussion

This finding is not surprising. After all, excitement around the approval of the HIV self-test was deeply rooted in its promise to aid individuals in overcoming historic barriers to testing ((11)). These include, but are not limited to, geographic distance, conflicting hours of operation and experiences of racism, transphobia, homophobia, stigma and/or other forms of discrimination in healthcare ((12)). Recent reports indicate that more than six million Canadians say that they do not have regular access to a primary care provider ((13)). This threatens to place undue stress on other services. A survey completed in 2020 found that 39% of Canadians visited an emergency department for an issue that could have been treated by a primary care provider ((14)). One such item that people have been seeking through emergency departments includes testing for sexual transmitted infections and HIV ((15)). It is within this environment that the uptake of, and the demographics of individuals ordering, HIV self-tests should be contextualized. Given historical barriers to services, compounded by the pandemic, the popularity of the HIV self-test may say more about the state of our primary care system than it does about the usefulness of the device itself. In other words, people were excited for HIV self-testing not because this was necessarily how they wanted to do HIV testing; rather, they simply wanted access to such testing (which was otherwise difficult for them to obtain).

In 2023, GetaKit.ca expanded its testing options to include testing for other sexually transmitted and blood-borne infections (STBBIs). Working in close partnership with public health units in Ontario, GetaKit.ca now offers testing for gonorrhea, chlamydia, syphilis, hepatitis C and HIV serology to participants who, in alignment with current clinical guidelines, meet the criteria for testing. If a participant lives in an area where full STBBI testing is available, they will be prompted to select the tests they would like to receive. Early findings indicate that while the number of orders that are being processed through the GetaKit.ca platform have increased over tenfold since STBBI testing became available (which has resulted in HIV testing nearly tripling), the demand for the HIV self-test has decreased. In other words, when other tests (including the option of serological testing for HIV) become readily available, participants not only choose full testing, but they also opt for serology over the HIV self-test. Perhaps it is unsurprising that, when given the option, people select the higher quality healthcare service.

In all cases, these findings are encouraging, indicating that GetaKit.ca can provide additional services to bridge the gap in testing created in the wake of the pandemic. For individuals, this means providing an easy and convenient access point for routine testers, as well as providing an option for others who may be unable or unwilling to attend an in-person appointment. For sexual health clinics, this means reducing pressure on already tight resources in a sector that has yet to fully recover from the impact of COVID-19 ((16)).

## Limitations

The success of GetaKit.ca comes with caveats. While the availability of STBBI testing through GetaKit.ca absolutely provides an opportunity to lessen the stress on brick-and-mortar clinics, redirects routine procedures to self-collection methods and offers judgment-free services, it should not be forgotten that these are necessary because our healthcare system is unable to meet the current demand for care. Again, the success of GetaKit.ca may speak more to the state of our healthcare system, rather than to the platform being ideal. There are other limitations that must be acknowledged; for example, the individuals who can access testing through digital platforms represent a subset of the at-risk populations who are able to overcome different barriers, such as digital and health literacy, access to stable internet and a fixed address for shipping. Moreover, results that necessitate follow-up testing, repeat testing or treatment still require that individuals interact with the healthcare system in its current state and there remain some communities who are unable or unwilling to do this. For some, this may mean reintroducing the original barriers, such as geographical distance, inconvenient hours of operation and experiences of racism and discrimination. We must therefore exercise caution when discussing the benefits of HIV self-testing, which is predominantly available online, lest those who are unable to navigate access (i.e., due to low digital literacy, limited access to stable internet, no fixed address) and have limited linkage to care pathways are left with fewer or no options for testing. Finally, at the time of writing, there exists no permanent public source of funding for HIV self-test distribution, which calls into question its long-term availability.

## Conclusion

Projects like GetaKit.ca, which offer health services in alignment with clinical guidelines with a focus on strong linkage to care pathways, can likely bridge a major gap in access to HIV testing (and to broader STI testing) for many. Participant preference for HIV serology over the HIV self-test when given the choice indicates that it is access to healthcare services, not to any one device, that attracts people. The success and sustainability of new testing devices and digital health services depend on strong primary care services to fulfill their mandate in offering high quality, low barrier healthcare for everyone. This means, as we continue to improve access to STBBI testing, we must also invest in our primary care system to enable providers to support new diagnoses identified through projects like GetaKit.ca and provide judgment-free care to individuals with complex care needs.
